# Incorporating single-arm studies in meta-analysis of randomised controlled trials: a simulation study

**DOI:** 10.1186/s12874-021-01301-1

**Published:** 2021-06-03

**Authors:** Janharpreet Singh, Keith R. Abrams, Sylwia Bujkiewicz

**Affiliations:** 1 Biostatistics Research Group, Department of Health Sciences, University of Leicester, Leicester, UK; 2 Centre for Health Economics, University of York, York, UK

**Keywords:** Evidence synthesis, Real world data, Single-arm studies, Bayesian hierarchical methods, Meta-analysis, Arm-based methods

## Abstract

**Background:**

Use of real world data (RWD) from non-randomised studies (e.g. single-arm studies) is increasingly being explored to overcome issues associated with data from randomised controlled trials (RCTs). We aimed to compare methods for pairwise meta-analysis of RCTs and single-arm studies using aggregate data, via a simulation study and application to an illustrative example.

**Methods:**

We considered contrast-based methods proposed by Begg & Pilote (1991) and arm-based methods by Zhang *et al* (2019). We performed a simulation study with scenarios varying (i) the proportion of RCTs and single-arm studies in the synthesis (ii) the magnitude of bias, and (iii) between-study heterogeneity. We also applied methods to data from a published health technology assessment (HTA), including three RCTs and 11 single-arm studies.

**Results:**

Our simulation study showed that the hierarchical power and commensurate prior methods by Zhang *et al* provided a consistent reduction in uncertainty, whilst maintaining over-coverage and small error in scenarios where there was limited RCT data, bias and differences in between-study heterogeneity between the two sets of data. The contrast-based methods provided a reduction in uncertainty, but performed worse in terms of coverage and error, unless there was no marked difference in heterogeneity between the two sets of data.

**Conclusions:**

The hierarchical power and commensurate prior methods provide the most robust approach to synthesising aggregate data from RCTs and single-arm studies, balancing the need to account for bias and differences in between-study heterogeneity, whilst reducing uncertainty in estimates. This work was restricted to considering a pairwise meta-analysis using aggregate data.

**Supplementary Information:**

The online version contains supplementary material available at (10.1186/s12874-021-01301-1).

## Background

Health technology assessment (HTA) decision-makers, such as the National Institute for Health and Care Excellence in England and Wales, recommend new health technologies for reimbursement based on cost-effectiveness. They consider the clinical effectiveness of a technology against comparators, estimated by a meta-analysis of studies conducted in similar patient populations recording a common outcome measure [1]. A randomised controlled trial (RCT) provides the best evidence of relative effectiveness because random treatment allocation minimises participant selection bias between arms [2]. However, decision-makers may consider observational evidence (e.g. single-arm studies) when, for example, a technology has received accelerated regulatory approval [3]. This suggests a need to develop meta-analysis methods which can combine randomised and non-randomised studies, whilst addressing issues in non-randomised data. Bayesian methods provide a flexible approach for combining data from different sources, and can be implemented via Markov chain Monte Carlo (MCMC) sampling which aids probablistic decison-making in HTA [4].

A number of methods have been proposed for pairwise meta-analysis of RCTs and single-arm studies using aggregate data, which make different assumptions regarding data variability. Begg & Pilote [5] proposed a method under a frequentist framework, which assumes exchangeability for baseline treatment effects and a common relative treatment effect. The method does not distinguish between RCTs and single-arm studies, but can be extended to account for bias in single-arm data. In this context, bias refers to the systematic difference between data from RCTs and single-arm studies. Zhang *et al* [6] proposed several methods under a Bayesian framework, which assume exchangeability for treatment effects on each arm. The methods distinguish between RCTs and single-arm studies, by assuming correlation between RCT arms and differences in between-study heterogeneity. Some of the methods use single-arm data to inform prior distributions for model parameters. Although Zhang *et al* performed a simulation study to compare the relative performance between their methods [6], there has been no comparison of these methods with the methods by Begg & Pilote. Other methods, which are not considered here, use study-matching or individual participant data (IPD) to perform a network meta-analysis (NMA) of RCTs and single-arm studies. Schmitz *et al* [7] proposed a method using aggregate data on patient characteristics to match single-arm studies with similar patient samples, and perform a NMA of RCTs and the matched studies. Thom *et al* [8] proposed a method which assumes exchangeability for baseline treatment effects, and uses IPD to adjust for covariates.

In this paper, we focus on the methods proposed by Begg & Pilote [5] and Zhang *et al* [6], which combine data from RCTs and single-arm studies at the aggregate level. The two sets of meta-analytic methods are contrast-based and arm-based methods, respectively. We aim to compare both sets of methods to investigate how these different approaches, as well as a number of other specific assumptions, affect their relative performance. We compare the methods in an extensive simulation study, building on the simulation study by Zhang *et al* [6]. We evaluate performance under a number of scenarios varying the proportion of RCTs and single-arm studies in the synthesis, the magnitude of bias between data from RCTs and single-arm studies, and differences in between-study heterogeneity across RCTs and single-arm studies.

### Illustrative example: dataset

**Fig. 1:**
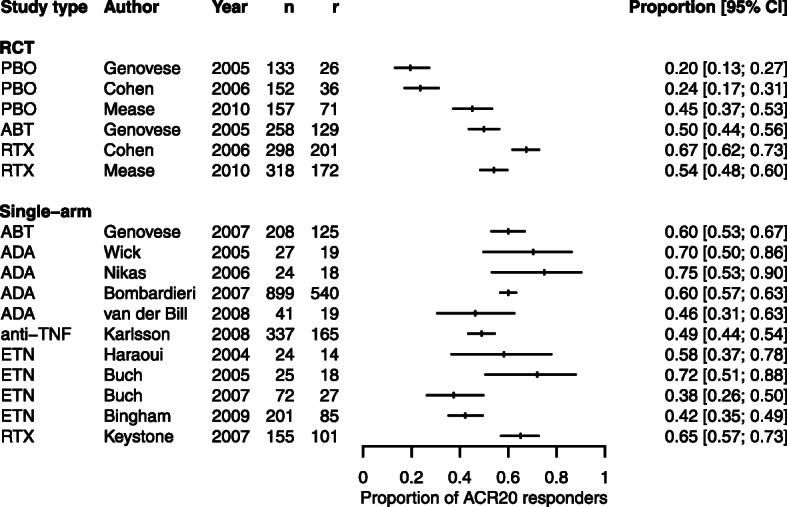
An arm-level forest plot of the proportion of ACR20 responders in each study

Rheumatoid arthritis (RA) is a chronic auto-immune condition causing joint inflammation, which can be treated by a number of biologic disease-modifying anti-rheumatic drugs (bDMARDs); adalimumab (ADA), etanercept (ETN), infliximab (IFX), abatacept (ABT), and rituximab (RTX) [9]. The treatment response can be assessed by using the American College of Rheumatology (ACR) response criteria, where ACR20 represents a 20% improvement in symptoms [10]. Malottki *et al* assessed the clinical effectiveness of bDMARDs in a HTA [11], identifying three RCTs and 11 single-arm studies for which data were available on the ACR20 outcome. Figure 1 shows a forest plot illustrating the arm-level proportions of ACR20 responders in each study. The plot does not suggest a systematic bias between data from RCTs and single-arm studies on the bDMARD arm.

There are three RCTs in which participants have been assigned a placebo or a bDMARD, and 11 single-arm studies in which participants were assigned a bDMARD. We select the three placebo arms as the baseline, so that *π*_1_ represents the marginal response probability for participants assigned a placebo, and *π*_2_ represents the marginal response probability for participants assigned a bDMARD. Thus, the odds ratio represents the increase in odds of achieving a ACR20 response for participants given a bDMARD versus placebo.

## Methods

### Methods for meta-analysis of RCTs and single-arm studies using aggregate data

In this section, we describe the methods by Begg & Pilote [5] and by Zhang *et al* [6] under a Bayesian framework. Although Begg & Pilote introduced methods under a frequentist framework, they are adapted here for Bayesian implementation to ensure a fair comparison between both sets of methods. For consistency, and to enable a direct comparison of all methods, we adapt the methods by Begg & Pilote to a dichotomous outcome. We consider a pairwise meta-analysis, with *n* RCTs assessing treatments one and two, *m* single-arm studies assessing treatment one, and *l* single-arm studies assessing treatment two. We let *i*=1,...,*n* index RCTs, *i*=*n*+1,...,*n*+*m* indexes single-arm studies on arm 1, and *i*=*n*+*m*+1,...,*n*+*m*+*l* indexes single-arm studies on arm 2. Here, we describe first the methods introduced by Begg & Pilote, and then the methods introduced by Zhang et al. The first set of methods parametrise treatment effect contrasts, whilst the latter parametrise treatment effects on each arm. For clarity, we define notation as we introduce each method and attempt to use the original symbols where possible. For the methods by Begg & Pilote, we begin by describing the original method (BP), and then describe the bias-adjusted (BPbias) and random-effects (BPrandom) methods by showing how they build-on the BP method. For the methods by Zhang et al, we begin by describing the bivariate generalised linear mixed-effects model (BGLMM) I method, and then describe the BGLMM II, hierarchical power prior (HPP) and hierarchical commensurate prior (HCP) methods by showing how they build-on the BGLMM I method. We then describe how marginal response probabilities are calculated for each method.

#### Begg & Pilote (BP) original method

By adapting the method by Begg & Pilote (BP) to Binomial data, it assumes that in each arm of study *i* the number of responders follows a Binomial distribution

1 $$ \begin{aligned} &r_{1i} \sim Bin\left(n_{1i}, p_{1i}\right), \qquad i=1,..., n + m; \\ &r_{2i} \sim Bin\left(n_{2i}, p_{2i}\right), \qquad i=1,..., n; \quad i = n + m + 1,..., n + m + l; \end{aligned}  $$

where *n*_1*i*_,*n*_2*i*_ are the numbers of participants on arms one and two, respectively, and *p*_1*i*_,*p*_2*i*_ are the response probabilities on arms one and two, respectively. The response probability in each arm is transformed onto the linear predictor scale using a suitable link function *g*()

2 $$ \begin{aligned} &g\left(p_{1i}\right) = \theta_{i}, \qquad i=1,..., n + m; \\ &g\left(p_{2i}\right) = \theta_{i} + \delta, \qquad i=1,..., n; \quad i = n + m + 1,..., n + m + l; \end{aligned}  $$

where *θ*_*i*_ represents the baseline treatment effect (i.e. the treatment effect in arm one) in study *i*, and *δ* represents the relative treatment effect (i.e. the treatment effect in arm two relative to arm one). Here, the relative treatment effect is assumed to be identical across all studies, whilst the baseline treatment effects are exchangeable (i.e. vary across studies according to a common distribution)

3 $$\begin{array}{*{20}l} \theta_{i} \sim N\left(\mu, \sigma^{2}\right), \qquad i=1,..., n + m + l; \end{array} $$

with mean *μ* and standard deviation *σ*. Suitably non-informative prior distributions can be placed on *μ* and *σ*; *μ*∼*N*(0,10^5^),*σ*∼*Γ*^−1^(10^−4^,10^−4^).

#### Begg & Pilote method with bias-adjustment (BPbias)

The bias-adjusted version of the BP method (BPbias) extends BP in Eq. (2) with the additional assumption that single-arm data are systematically biased relative to RCT data

4 $$\begin{array}{*{20}l} &g\left(p_{1i}\right) = \theta_{i} + \xi, \qquad i=n + 1,..., n + m; \\ &g\left(p_{2i}\right) = \theta_{i} + \delta + \eta, \qquad i = n + m + 1,..., n + m + l; \end{array} $$

where *ξ* (for arm one) and *η* (for arm two) represent bias in the single-arm data. The bias is assumed to be common across single-arm studies and suitably non-informative Normal prior distributions can be placed on the bias parameters; *ξ*∼*N*(0,10^5^) and *η*∼*N*(0,10^5^).

#### Begg & Pilote method with random effects (BPrandom)

The BP method with random effects (BPrandom) extends BP in Eq. (2) by assuming exchangeable relative treatment effects

5 $$ \begin{aligned} &g\left(p_{1i}\right) = \theta_{i}, \qquad i=1,..., n + m; \\ &g\left(p_{2i}\right) = \theta_{i} + \delta_{i}, \qquad i=1,..., n; \quad i = n + m + 1,..., n + m + l; \end{aligned}  $$

where *δ*_*i*_ are the relative treatment effects assumed to follow a Normal distribution

6 $$ \begin{aligned} \delta_{i} \sim N\left(d, \tau^{2}\right), \qquad i=1,..., n; \quad i = n + m + 1,..., n + m + l; \end{aligned}  $$

with mean *d* and standard deviation *τ*. Suitably non-informative prior distributions can be placed on *d* and *τ*; *d*∼*N*(0,10^5^),*τ*∼*Γ*^−1^(10^−4^,10^−4^).

#### Bivariate generalised linear mixed effects models (BGLMM) I & II

The first method proposed by Zhang *et al* is bivariate generalised linear mixed-effects model (BGLMM) I, which assumes a Binomial likelihood for the arm-level data as formulated in Eq. (1). In contrast to Begg & Pilote, Zhang *et al* model the treatment effect in each arm of study *i*. For RCTs, the method assumes data are correlated between arms

7 $$\begin{array}{*{20}l} &g\left(p_{1i}\right) = \mu_{1} + \nu_{1i}, \\ &g\left(p_{2i}\right) = \mu_{2} + \nu_{2i}, \\ &\left(\nu_{1i}, \nu_{2i}\right) \sim N\left(\mathbf{0}, \mathbf{\Sigma}\right), \qquad i=1,..., n; \\ &\mathbf{\Sigma} = \left(\begin{array}{cc} \sigma_{1}^{2} & \rho \sigma_{1} \sigma_{2} \\ \rho \sigma_{1} \sigma_{2} & \sigma_{2}^{2} \end{array}\right) \end{array} $$

where *μ*_1_ and *μ*_2_ represent the mean treatment effect in each arm, whilst (*ν*_1*i*_,*ν*_2*i*_) are assumed to follow a bivariate Normal distribution with covariance matrix *Σ*, which accounts for between-study heterogeneity across RCTs on each arm and correlation between arms. Non-informative Normal prior distributions can be placed on the mean treatment effects; *μ*_1_∼*N*(0,10^5^),*μ*_2_∼*N*(0,10^5^). An inverse-Wishart prior distribution can be placed on the covariance matrix; *Σ*∼*W*^−1^(*R*,2), where *R* is a 2×2 scale matrix with diagonal elements equal to 1 and off-diagonal elements equal to 0.005. This prior distribution is weakly informative on both the correlation and standard deviation parameters, but correctly implies that the population-averaged treatment-specific event probabilities range from 0 to 1. The method assumes the same mean treatment effects *μ*_1_ and *μ*_2_ for single-arm studies

8 $$ \begin{aligned} &g\!\left(p_{1i}\right) = \mu_{1}\! + \nu_{3i}, \quad \nu_{3i} \sim N\!\left(0, \sigma_{3}^{2}\right) \qquad i=n + 1,..., n + m; \\ &g\!\left(p_{2i}\right) = \mu_{2}\! + \nu_{4i}, \quad \nu_{4i} \sim N\!\left(0, \sigma_{4}^{2}\right) \qquad i=n + m + 1,..., n + m + l; \end{aligned}  $$

where *ν*_3*i*_ and *ν*_4*i*_ are each assumed to follow a univariate Normal distribution to account for the between-study heterogeneity across single-arm studies on each arm. Similar to Zhang et al, we place inverse-Gamma prior distributions on the standard deviation parameters; *σ*_3_∼*Γ*^−1^(10^−4^,10^−4^) and *σ*_4_∼*Γ*^−1^(10^−4^,10^−4^).

The BGLMM I method can be modified in Eq. (8) to assume different mean treatment effects *μ*_3_ and *μ*_4_ for single-arm studies

9 $$ \begin{aligned} &g\left(p_{1i}\right) =\! \mu_{3} + \nu_{3i}, \quad \nu_{3i} \sim N\left(0, \sigma_{3}^{2}\right) \qquad i=\! n \! + 1,..., n + m; \\ &g\left(p_{2i}\right) =\! \mu_{4} + \nu_{4i}, \quad \nu_{4i} \sim N\left(0, \sigma_{4}^{2}\right) \qquad i=\! n \! + m + 1,..., n + m + l; \end{aligned}  $$

Non-informative Normal prior distributions can be placed on the mean treatment effects; *μ*_3_∼*N*(0,10^5^),*μ*_4_∼*N*(0,10^5^), which can themselves be applied to inform prior distributions for *μ*_1_ and *μ*_2_ in a two-step method. First, the model specified in Eq. (9) is fit to the single-arm data to estimate posterior distributions for *μ*_3_ and *μ*_4_, from which posterior median and standard deviation estimates are obtained. Then, the model specified in Eq. (7) is fit to the RCT data, with informative prior distributions (based on the extracted estimates) placed on the mean treatment effects; $\mu _{1} \sim N\left (\hat {\mu _{3}}, \hat {\tau _{1}}^{2}\right), \mu _{2} \sim N\left (\hat {\mu _{4}}, \hat {\tau _{2}}^{2}\right)$. This modified-version of the BGLMM I method is labelled BGLMM II.

#### Hierarchical power prior (HPP)

The hierarchical power prior (HPP) method extends the BGLMM I method in Eq. (1) by raising the likelihood functions *L*(*p*_1*i*_) and *L*(*p*_2*i*_) for the single-arm studies to a power between zero and one

10 $$ \begin{aligned} &L\left(p_{1i}\right) = \left(p_{1i}^{r_{1i}}\left(1-p_{1i}\right)^{n_{1i}-r_{1i}}\right)^{\alpha_{1}}, \qquad i = n + 1,..., n + m; \\ &L\left(p_{2i}\right) = \left(p_{2i}^{r_{2i}}\left(1-p_{2i}\right)^{n_{2i}-r_{2i}}\right)^{\alpha_{2}}, \qquad i = n + m + 1,..., n + m + l; \end{aligned}  $$

where *α*_1_ and *α*_2_ represent the power parameters for each arm. To allow flexibility in down-weighting the single-arm data, Beta prior distributions can be placed on the power parameters; *α*_1_∼*β*(10,1),*α*_2_∼*β*(10,1). A *β*(10,1) prior has mean 0.91 and a 95% credible interval ranging from 0.69 to 0.99, which indicates a moderate-to-strong similarity between single-arm studies and RCTs, and provides a modest down-weighting [6].

#### Hierarchical commensurate prior (HCP)

The hierarchical commensurate prior (HCP) method assumes different mean treatment effects for RCTs and single-arm studies (described by Eqs. (7) and (9)), and places Normal prior distributions on *μ*_1_ and *μ*_2_ informed by the single-arm data;

11 $$\begin{array}{*{20}l} &\mu_{1} \sim N\left(\mu_{3}, \frac{1}{\tau_{1}^{2}}\right) \\ &\mu_{2} \sim N\left(\mu_{4}, \frac{1}{\tau_{2}^{2}}\right) \end{array} $$

where *τ*_1_ and *τ*_2_ are commensurability parameters representing agreement between data from RCTs and single-arm studies. Similar to Zhang et al, we place Gamma prior distributions on each parameter; *τ*_1_∼*Γ*(10^−3^,10^−3^),*τ*_2_∼*Γ*(10^−3^,10^−3^). For small parameter values, the variance of the single-arm data is inflated and the contribution to *μ*_1_ and *μ*_2_ is down-weighted. As parameter values approach zero, only RCT data contribute in estimating *μ*_1_ and *μ*_2_, whilst single-arm data are ignored. As the parameter values approach infinity, data from RCTs and single-arm studies contribute equally in estimating *μ*_1_ and *μ*_2_.

### Marginal response probabilities

The methods described above for a dichotomous outcome model the response probability in each arm, based on the numbers of participants and responders (described by Eq. (1)), and use a link function *g*() to transform the response probability onto the linear predictor scale where treatment effects are additive. A logit or probit link function can be used for meta-analysis with Binomial data [4], although the logit link is often favoured in the published literature as the relative treatment effects are easier to interpret on the log odds ratio scale. The methods proposed by Zhang *et al* do not parametrise relative treatment effects, and instead they recommend using the probit link *Φ*^−1^() and then calculating the marginal response probability in each arm

12 $$\begin{array}{*{20}l} &\pi_{1} = \Phi\left(\frac{\mu_{1}}{\sqrt{1 + \sigma_{1}^{2}}}\right) \\ &\pi_{2} = \Phi\left(\frac{\mu_{2}}{\sqrt{1 + \sigma_{2}^{2}}}\right) \end{array} $$

where *Φ* is the cumulative distribution function for the standard Normal distribution. The marginal response probabilities can be used to calculate a marginal odds ratio *OR*_21_=*π*_2_(1−*π*_1_)/*π*_1_(1−*π*_2_). We implement the methods proposed by Begg & Pilote using a probit link to allow a direct comparison of all methods. For the BP and BPbias methods, we obtain the marginal response probabilities using Eq. (13)

13 $$\begin{array}{*{20}l} &\pi_{1} = \Phi\left(\frac{\mu}{\sqrt{1 + \sigma^{2}}}\right) \\ &\pi_{2} = \Phi\left(\frac{\mu + \delta}{\sqrt{1 + \sigma^{2}}}\right) \end{array} $$

and for the BPrandom method using Eq. (14)

14 $$\begin{array}{*{20}l} &\pi_{1} = \Phi\left(\frac{\mu}{\sqrt{1 + \sigma^{2}}}\right) \\ &\pi_{2} = \Phi\left(\frac{\mu + d}{\sqrt{1 + (\sigma + \tau)^{2}}}\right) \end{array} $$

### Summary of methods

In this section, we have described the details of each method (including suitable prior distributions) and the corresponding marginal response probabilities (WinBUGS code used to fit each of the methods is provided in Appendix D). We note that the methods proposed by Zhang *et al* reduce to the model described by Eq. (7) when applied to RCT data only (*i*=1,...,*n*), which we label BGLMM ^∗^. Similarly, the BP and BPbias methods reduce to the model described by Eqs. (2) and (3) when applied to RCT data only, which we label BP ^∗^. We label the BPrandom method applied to RCT data only as BPrandom ^∗^.

### Simulation study: methods

In this section, we report aims, data-generation methods, estimands, methods, and performance measures for the simulation study, as recommended by Morris *et al* [12]. The simulation study aimed to compare the performance of the methods described previously, under a number of scenarios varying the proportion of RCTs and single-arm studies in the synthesis, the magnitude of bias between data from RCTs and single-arm studies, and differences in between-study heterogeneity across RCTs and single-arm studies. We aimed to build-on the simulation study performed by Zhang *et al* [6], where the estimands were the marginal response probability in each arm *π*_1_ and *π*_2_. We evaluated performance by calculating coverage, mean-square error (MSE), and mean change in 95% credible interval length (CrIL). The latter measures the average change in CrIL when a method is applied to *RCT and single-arm data* versus *RCT data only*. The methods were implemented via MCMC sampling in the WinBUGS software, using a burn-in of 20,000 iterations and 100,000 iterations for posterior estimation [13].

#### Data-generation methods

As in Zhang *et al* [6], we let *n* represent the number of RCTs assessing treatments one and two, *m* - the number of single-arm studies assessing treatment one, and *l* - the number of single-arm studies assessing treatment two. We let *i* denote study, and set the total number of studies in a dataset *n*+*m*+*l*=30. The data were simulated based-on the BGLMM I method, modified to assume bias in the single-arm data. The steps taken to simulate a dataset were as follows. For RCT data, we specified values for the between-study heterogeneity on each arm (*σ*_1_ and *σ*_2_) and correlation between arms (*ρ*) to obtain the covariance matrix (*Σ*) to simulate *v*_1*i*_ and *v*_2*i*_

$$\begin{array}{*{20}l} &\mathbf{\Sigma} = \left(\begin{array}{cc} \sigma_{1}^{2} & \rho \sigma_{1} \sigma_{2} \\ \rho \sigma_{1} \sigma_{2} & \sigma_{2}^{2} \end{array}\right) \\ &\left(\nu_{1i}, \nu_{2i}\right) \sim N\left(\mathbf{0}, \mathbf{\Sigma}\right), \qquad i = 1,..., n; \end{array} $$

We assigned values to the mean treatment effect in each arm (*μ*_1_ and *μ*_2_), and applied the simulated *v*_1*i*_ and *v*_2*i*_ to obtain response probabilities on each arm

$$\begin{array}{*{20}l} &p_{1i} = \Phi\left(\mu_{1} + \nu_{1i}\right), \\ &p_{2i} = \Phi\left(\mu_{2} + \nu_{2i}\right), \qquad i=1,..., n; \end{array} $$

where *Φ* is the cumulative distribution function for the standard Normal distribution. We set the number of participants to 100 in each arm of study *i*, and applied the response probabilities (*p*_1*i*_ and *p*_2*i*_) to sample the number of responders (*r*_1*i*_ and *r*_2*i*_) from a Binomial distribution

$$\begin{array}{*{20}l} &r_{1i} \sim Bin\left(100, p_{1i}\right), \\ &r_{2i} \sim Bin\left(100, p_{2i}\right), \qquad i=1,..., n; \end{array} $$

For single-arm data, we specified values for the between-study heterogeneity on each arm (*σ*_3_ and *σ*_4_) to simulate *v*_3*i*_ and *v*_4*i*_

$$\begin{array}{*{20}l} &\nu_{3i} \sim N\left(0, \sigma_{3}^{2}\right) \qquad i=n + 1,..., n + m; \\ &\nu_{4i} \sim \left(0, \sigma_{4}^{2}\right) \qquad i=n + m + 1,..., n + m + l; \end{array} $$

We defined values for the bias in each arm (*ξ* and *η*), and applied the simulated *v*_3*i*_ and *v*_4*i*_ together with the mean treatment effects (*μ*_1_ and *μ*_2_), to obtain the response probabilities on each arm

$$\begin{array}{*{20}l} &p_{1i} = \Phi\left(\mu_{1} + \nu_{3i} + \xi\right), \qquad i=n + 1,..., n + m; \\ &p_{2i} = \Phi\left(\mu_{2} + \nu_{4i} + \eta\right), \qquad i=n + m + 1,..., n + m + l; \end{array} $$

We applied the response probabilities (*p*_1*i*_ and *p*_2*i*_) to sample the numbers of responders (*r*_1*i*_ and *r*_2*i*_) from a Binomial distribution

$$\begin{array}{*{20}l} &r_{1i} \sim Bin\left(100, p_{1i}\right), \qquad i=n + 1,..., n + m; \\ &r_{2i} \sim Bin\left(100, p_{2i}\right), \qquad i=n + m + 1,..., n + m + l; \end{array} $$

The data were simulated under a number of scenarios adapted from scenario 1 (S1), where the number of RCTs *n*=15 and the number of single-arm studies on each arm *m*=10 and *l*=5. The magnitude of bias for single-arm studies *ξ*=0.2 and *η*=0.4, and between-study heterogeneity parameters *σ*_1_=0.6,*σ*_2_=0.7,*σ*_3_=0.8,*σ*_4_=1. Due to lack of randomisation, single-arm data are at a higher risk of bias compared to randomised data, so we assume a systematic difference (i.e. parameters *ξ*=0.2 and *η*=0.4) and larger between-study heterogeneity (i.e. parameters *σ*_3_=0.8 and *σ*_4_=1.0). For all scenarios, the mean treatment effects were set to *μ*_1_=0.4 and *μ*_2_=1.1, and correlation was *ρ*=0.6. We arrange the scenarios into four groups, where in each group the scenarios focus on varying a common set of parameter values. In group one, S1-5, the number of RCTs gradually decreases (from *n*=15 to *n*=1). This was intended to clearly demonstrate the performance of the methods in scenarios where there is little randomised evidence (i.e. S4 and S5, where *n*=3 and *n*=1) compared to scenarios where there is relatively substantial randomised evidence (i.e. S1, where *n*=15). In group two, [S6, S1, S7-9], the bias gradually increases (from *ξ*=0,*η*=0 to *ξ*=0.8,*η*=1). In group three, [S10-12, S6], the between-study heterogeneity for single-arm data gradually increases (from *σ*_3_=0.1,*σ*_4_=0.3 to *σ*_3_=0.8,*σ*_4_=1), with zero bias (*ξ*=0,*η*=0). In group four, [S13-15, S1], the between-study heterogeneity for the single-arm data gradually increases (from *σ*_3_=0.1,*σ*_4_=0.3 to *σ*_3_=0.8,*σ*_4_=1), with non-zero bias (*ξ*=0.2,*η*=0.4). A full description of the parameter values specified in each scenario is provided in Table A.1 (Appendix A).

## Results

### Illustrative example: results

Table 1 presents posterior median estimates (and 95% credible intervals) for *π*_1_,*π*_2_, and the marginal odds ratio. The results are presented separately for analysis of *RCT and single-arm data* versus analysis of *RCT data only*. For the latter analysis, a random-effects meta-analysis (REMA) [14] and fixed-effect meta-analysis (FEMA) were also implemented. The odds ratio estimates range from 2.53 to 3.4, suggesting participants assigned a bDMARD versus placebo were more than twice as likely to achieve an ACR20 response. However, only the contrast-based methods show CrIs greater than one. There is a reduction in uncertainty when methods are applied to include single-arm studies in the synthesis, and the arm-based methods show a greater reduction in CrIL for the odds ratio (between 23-49%) compared to the contrast-based methods (between 17-22%). Table 1 includes estimates for the deviance information criterion (DIC), which provides a measure of model fit whilst penalising model complexity [15]. The BP method has the highest DIC value and is the simplest method in terms of model parameters.

**Table 1:** Posterior median estimates and 95% credible intervals from application to illustrative example

Method	*π*_1_	*π*_2_	OR	DIC
BGLMM1	0.32 (0.14, 0.59)	0.55 (0.49, 0.62)	2.65 (0.84, 7.68)	104.10 (94.93, 118.30)
BGLMM2	0.33 (0.15, 0.62)	0.55 (0.49, 0.62)	2.53 (0.74, 7.31)	
HPP	0.32 (0.13, 0.60)	0.56 (0.40, 0.72)	2.73 (0.74, 10.64)	
HCP	0.32 (0.13, 0.64)	0.56 (0.35, 0.72)	2.60 (0.61, 9.38)	104.20 (95.07, 118.40)
BP	0.30 (0.23, 0.37)	0.56 (0.51, 0.62)	3.06 (2.42, 3.89)	131.40 (122.70, 144.90)
BPbias	0.30 (0.20, 0.44)	0.57 (0.45, 0.69)	3.06 (2.41, 3.93)	131.10 (122.60, 144.90)
BPrandom	0.30 (0.15, 0.53)	0.55 (0.29, 0.77)	2.74 (1.05, 6.78)	103.90 (94.82, 118.40)
**RCT data only**				
BGLMM ^∗^	0.32 (0.13, 0.61)	0.56 (0.28, 0.78)	2.64 (0.51, 13.32)	39.08 (34.99, 48.09)
BP ^∗^	0.30 (0.19, 0.49)	0.57 (0.40, 0.71)	3.00 (2.00, 3.89)	65.84 (62.91, 73.80)
BPrandom ^∗^	0.31 (0.15, 0.58)	0.56 (0.26, 0.81)	2.76 (0.95, 7.85)	39.59 (35.11, 49.61)
REMA			3.40 (0.82, 14.02)	39.17 (35.02, 48.34)
FEMA			3.18 (2.49, 4.08)	65.95 (63.07, 73.78)

### Simulation study: results

In this section, we present the simulation study results for each method in terms of coverage, MSE and mean change in CrIL. We illustrate the results for each scenario group with a line plot of the performance measures for each estimand.

#### Scenarios S1-5

**Fig. 2:**
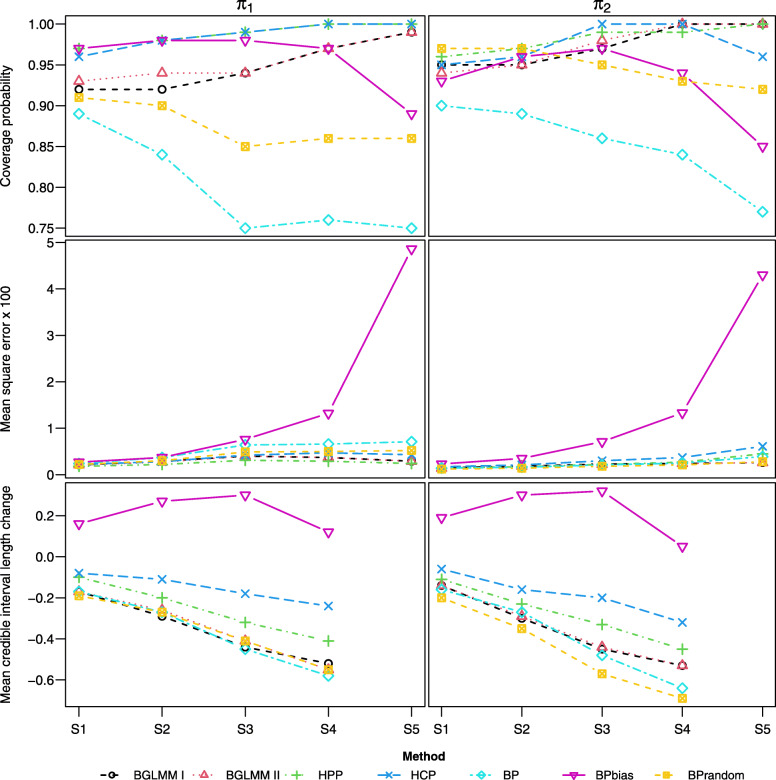
Coverage, MSE, and mean change in CrIL for each estimand, across scenarios S1-5 where the number of RCTs is gradually decreased

Across scenarios S1-5, the proportion of RCTs gradually decreases (from *n*=15 to *n*=1), whilst the total number of studies remains fixed (*n*+*m*+*l*=30). The results for these scenarios are presented in Fig. 2. The HPP and HCP methods, which both down-weight the single-arm data, perform relatively strongly with over-coverage (i.e. coverage above the nominal value 0.95) and small MSE for both estimands. This suggests that down-weighting the single-arm data can mitigate the effect of bias. The BPbias method, which includes a parameter on each arm to account for bias, performs strongly in S1-2 where there is a significant number of RCTs (*n*=15 and *n*=12). However, its performance drops-off and MSE is much larger in S4-5 where there are few RCTs (*n*=3 and *n*=1). This suggests that it requires a significant number of both RCTs and single-arm studies to estimate bias. The BP method is naive to study-design, and shows under-coverage for all scenarios, which worsens as the proportion of RCTs decreases. All methods, aside from BPbias, provide a reduction in uncertainty when including versus excluding single-arm data.

In scenarios S1-5, data were simulated using the BGLMM I method, potentially favouring arm-based methods over contrast-based methods. We performed a sensitivity analysis to further explore this, where data were simulated using the BPrandom method. We label these scenarios S 1^∗^- 5^∗^, and the results are presented in Figure B.1 (Appendix B). The HCP and HPP methods performed strongly across S1-5, and maintain their performance in S 1^∗^- 5^∗^ with over-coverage and relatively small MSE. The performance of BPbias in S 1^∗^- 5^∗^ mirrors its performance in S1-5, with a significant decrease in coverage and increase in MSE occurring in S 5^∗^. The BP and BPrandom methods show a reduction in under-coverage and MSE, perhaps because their assumptions are now better aligned with the data-generating method (e.g. common between-study heterogeneity across all studies). In contrast, the BGLMM I and II methods show a reduction in coverage and a small increase in MSE, because their assumptions are not as aligned to the data-generating method. The impact of between-study heterogeneity is explored further in scenarios [S10-12, S6] and [S13-15, S6].

#### Scenarios [S6, S1, S7-9]

**Fig. 3:**
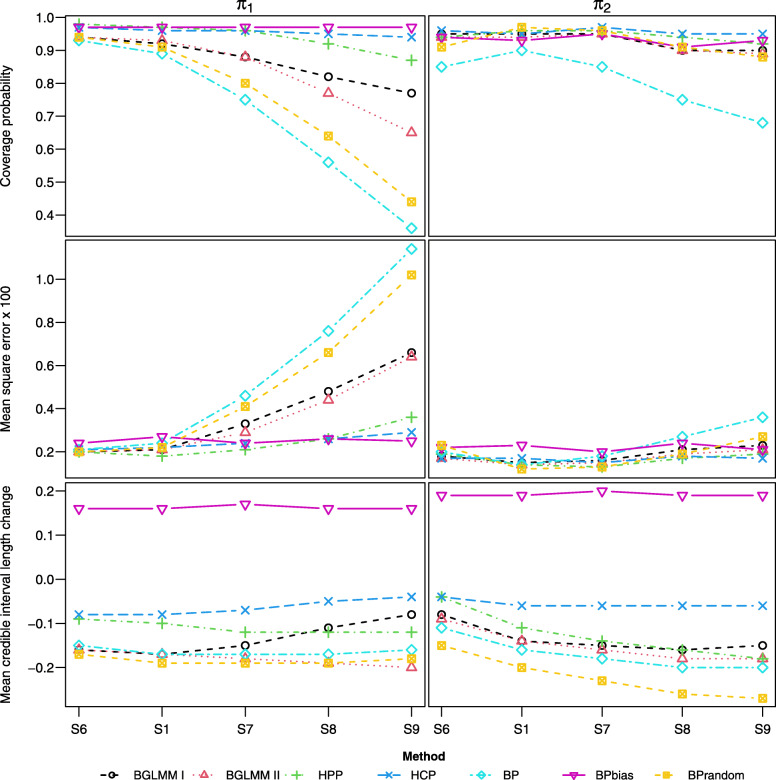
Coverage, MSE, and mean CrIL change for each estimand, across scenarios [S6, S1, S7-9] where bias in each arm gradually increases

Across scenarios [S6, S1, S7-9], the magnitude of bias in each arm gradually increases (from *ξ*=0,*η*=0 to *ξ*=0.8,*η*=1). The results for these scenarios are presented in Fig. 3. The BPbias method shows consistent over-coverage and small MSE, but does not offer any reduction in uncertainty when including single-arm studies. The HCP and HPP methods maintain coverage close to the nominal value and small MSE, whilst offering a consistent reduction in uncertainty. They only show a drop in performance in S9, where there is relatively large bias in single-arm data. The BP and BPrandom methods are naive to study-design and show reduction in uncertainty, but a steep decrease in coverage and increase in MSE as the bias is increased. The drop in performance is worse for *π*_1_ than *π*_2_, perhaps because there are more single-arm studies on arm one (*m*=10) than arm two (*l*=5). The BGLMM I and II methods do not account for bias in the single-arm data, but show a more gradual decrease in coverage and increase in MSE.

#### Scenarios [S10-12, S6]

**Fig. 4:**
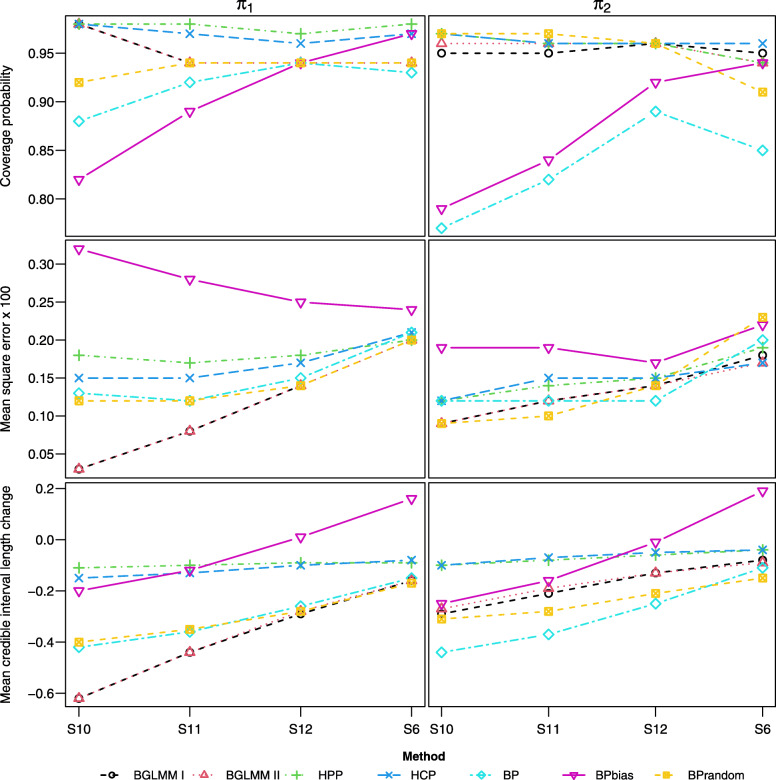
Coverage, MSE, and mean CrIL change for each estimand, across scenarios [S10-12, S6] where the between-study heterogeneity for single-arm studies gradually increases with zero bias

Across scenarios [S10-12, S6], between-study heterogeneity in single-arm studies on each arm gradually increases (from *σ*_3_=0.1,*σ*_4_=0.3 to *σ*_3_=0.8,*σ*_4_=1) but remains fixed in RCTs (*σ*_1_=0.6,*σ*_2_=0.7), and there is zero bias (*ξ*=0,*η*=0). Figure 4 presents the results for these scenarios. The BPbias method shows significant under-coverage and large MSE in S10 where the single-arm studies have much lower between-study heterogeneity compared to RCTs, but still provides some reduction in uncertainty. The BP and BPrandom methods show less under-coverage and much smaller MSE, whilst providing a greater reduction in uncertainty. The BGLMM I and II methods show over-coverage and little MSE, whilst providing the greatest reduction in uncertainty. As the between-study heterogeneity is increased, all methods show a decrease for the reduction in uncertainty, but the HCP and HPP methods are impacted the least.

#### Scenarios [S13-15, S1]

**Fig. 5:**
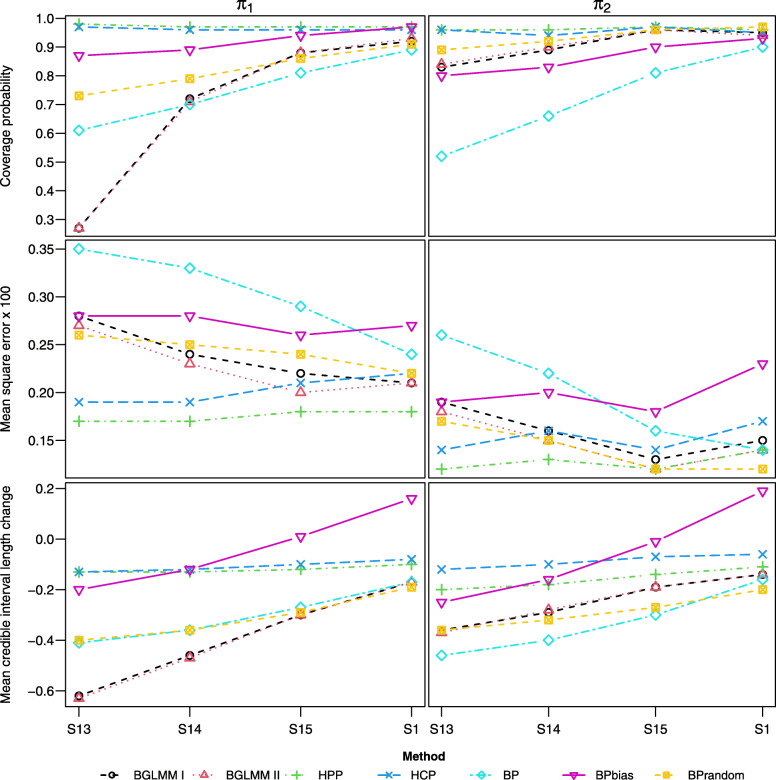
Coverage, MSE, and mean CrIL change for estimands, across scenarios [S13-15, S1] where the between-study heterogeneity for single-arm studies gradually increases with non-zero bias

In contrast to [S10-12, S6], scenarios [S13-15, S1] assume non-zero bias in the single-arm data (*ξ*=0.2,*η*=0.4), and the results are presented in Fig. 5. In S13, where single-arm data is much less uncertain than the RCT data, the BGLMM I and II methods show a significant reduction in uncertainty but large under-coverage and significant MSE. In comparison, the BP and BPrandom methods provide a more modest reduction in uncertainty but better coverage, although the BPrandom method shows large MSE. The BPbias method shows improvement compared to S10, but offers only a modest reduction in uncertainty which diminishes in [S14-15, S1]. The HCP and HPP methods, which down-weight single-arm data, show over-coverage and small MSE across the scenarios whilst maintaining a reduction in uncertainty.

## Discussion

In this paper, we aimed to compare methods proposed by Begg & Pilote and Zhang et al, for pairwise meta-analysis combining data from RCTs and single-arm studies using aggregate data. Based on our simulation study, we conclude that the HCP and HPP methods provide a consistent reduction in uncertainty when including single-arm data, whilst remaining robust to limited RCT data, bias, and differences in between-study heterogeneity across the two sets of data. Both methods achieve this by down-weighting the single-arm data, HPP through specification of a prior distribution, and HCP through estimating disagreement between the data from RCTs and single-arm studies. The BPbias method offers a simpler approach to mitigating bias, but requires a significant proportion of RCTs and single-arm studies in the synthesis. The BGLMM I and II methods provide a reduction in uncertainty, contingent upon little or no bias. Through our analysis of an illustrative example, we have shown that the methods can be used to combine data from RCTs and single-arm studies to achieve a significant reduction in uncertainty, compared to traditional meta-analysis of RCTs alone. We list below key recommendations in applying the methods for synthesis of RCTs and single-arm studies.

**Key recommendations:**

- The BP method is a parsimonious approach offering significant reduction in uncertainty (compared to the analysis of RCT data alone), when there is little or no bias and differences in between-study heterogeneity between the two types of data.
- The BGLMM I and II methods provide a significant reduction in uncertainty whilst accounting for differences in between-study heterogeneity, when there is little or no bias.
- The HPP method allows for down-weighting single-arm data, and remains robust to limited RCT data and bias whilst providing a reduction in uncertainty.
- The HCP method provides a consistent reduction in uncertainty whilst accounting for disagreement between RCT and single-arm data, and also remains robust to limited RCT data.

In a traditional meta-analysis of RCTs aiming to estimate a pooled relative treatment effect [16], baseline treatment effects are allowed to vary independently to preserve randomisation in each arm and minimise bias. There has been discussion in the literature regarding arm-based and contrast-based approaches to meta-analysis. Hong *et al* have suggested arm-based methods can minimise bias when data are assumed to be missing in a particular arm [17]. In response, Dias & Ades have argued that arm-based models actually increase bias since they do not preserve randomisation [18]. A further exploration of constrast-based and arm-based models has been performed by White *et al* considering a NMA context [19]. The traditional meta-analysis approach is not feasible when seeking to combine RCTs and single-arm studies, because the single-arm studies lack a comparator arm to estimate a relative treatment effect. Consequently, exchangeability must be assumed on at least one arm to incorporate the single-arm studies. The methods by Begg & Pilote assume exchangeability on the designated baseline arm, whilst those by Zhang *et al* assume exchangeability on both arms. Thus, it may be beneficial to perform a sensitivity analysis using more than one method. Decision-makers can then consider the benefits offered by including the single-arm studies (e.g. reduction in uncertainty) versus the potential penalties (e.g. increased risk of bias), and whether the penalties have been mitigated by applying a suitable method.

Aside from application in HTA, the methods assessed here can also be useful in clinical settings, for instance, in early-phase cancer research. Phase II cancer trials assess treatment efficacy via randomised-controlled or single-arm study designs, where only the latter may be ethical for rare cancers [20]. Consequently, there may be data available from both RCTs and single-arm studies, which need to be synthesised to determine the feasibility of a phase III trial [21]. Thus, further methodological development for performing a meta-analysis (or NMA [22]) to combine data from different study designs is required.

In this paper, we have considered the case where only aggregate data are available, which limits the methods to rudimentary bias-adjustment. The methods, however, can be very useful in situations when there are no IPD available and RCT data are limited. Further research is required to explore methods adjusting for bias which is variable across studies, to account for differences in risk of bias due to study setting (e.g. single-centre versus multi-centre single-arm studies). When IPD are available, a more detailed adjustment for potential biases can be carried out, as there are a number of approaches available that can be applied to mitigate confounding when estimating causal treatment effects [23]. For example, availability of IPD allows for enhancing approaches for meta-regression, which is recommended to explore bias and heterogeneity in a synthesis of evidence [24]. Furthermore, we did not consider the methods recently proposed by Schmitz *et al* [7], which incorporate single-arm studies in NMA of RCTs. Although proposed under a NMA context, they can be adapted for pairwise meta-analysis. However, after matching single-arm studies based on covariate information, the models used to synthesise data are only applicable to two-arm studies. Including those methods in this simulation study would also require specifying a model from which to simulate data on covariates. Thus, the simulation study was restricted to the methods by Begg & Pilote and Zhang et al.

## Conclusions

We have performed an extensive comparison of methods proposed by Begg & Pilote and Zhang et al, for pairwise meta-analysis combining data from RCTs and single-arm studies using aggregate data. We conclude that those methods by Zhang *et al* (HCP and HPP), which use the single-arm data to define prior distribution for model parameters, provide a consistent reduction in uncertainty when including single-arm data, whilst remaining robust to data variability. The other methods considered here perform worse when there is limited RCT data (BPbias), significant bias (BGLMM I & II), and differences in between-studies heterogeneity across the two sets of data (BP and BPrandom). We hope this study is informative for researchers seeking to perform a pairwise meta-analysis of RCTs and single-arm studies using aggregate data. We have described the existing methods in detail under a Bayesian framework, and the methods’ advantages and disadvantages under a number of data scenarios.

## Supplementary Information

**Additional file 1** Supplementary material for: Incorporating single-arm studies in meta-analysis of randomised controlled trials: A simulation

## Data Availability

All data generated or analysed during this study are included in this published article [and its Supplementary information files]. Declarations

## Abbreviations

HTA: Health technology assessment
RCT: Randomised controlled trial
MCMC: Markov chain Monte Carlo
NMA: Network meta-analysis
BP: Begg & Pilote
BPbias: Begg & Pilote method with bias-adjustment
BPrandom: Begg & Pilote method with random effects
BGLMM: Bivariate generalised linear mixed effects model
HPP: Hierarchical power prior
HCP: Hierarchical commensurate prior
MSE: Mean square error
CrIL: Credible interval length
S: Scenario
RA: Rheumatoid arthritis
bDMARDs: Biologic disease-modifying anti-rheumatic drugs
ADA: Adalimumab
ETN: Etanercept
IFX: Infliximab
ABT: Abatacept
RTX: Rituximab
ACR: American College of Rheumatology
REMA: Random effects meta-analysis
FEMA: Fixed effects meta-analysis
DIC: Deviance information criterion
IPD: Individual participant data.
