# Supplementary material for: Incorporating single-arm studies in meta-analysis of randomised controlled trials: a simulation study
Source: BMC Med Res Methodol. 2021 Jun 3;21:114. doi: 10.1186/s12874-021-01301-1 (PMC8176581; doi:10.1186/s12874-021-01301-1)
Supplement: Supplementary file 1 — Additional file 1 Supplementary material for: Incorporating single-arm studies in meta-analysis of randomised controlled trials: A simulation [file 12874_2021_1301_MOESM1_ESM.pdf]

Supplementary material for: *Incorporating single-arm studies in meta-analysis of randomised controlled trials: A simulation study*

Janharpreet Singh*[1], Keith R. Abrams[1], Sylwia Bujkiewicz[1]
[1]*Department of Health Sciences, University of Leicester, Leicester, UK*

*Corresponding author: js929@leicester.ac.uk

Appendix A - Simulation scenarios

We conducted a simulation study under 15 scenarios, using the model described in Section 3.1 to simulate data for 1000 iterations in each scenario. Table A.1 lists the model parameter values in the model which varied across scenarios. Here, n represents the number of RCTs, m and l represent the number of single-arm studies on each arm, ξ and η represent bias in single-arm data on each arm, σ_1 and σ_2 represent the between-study heterogeneity for each RCT arm, and σ_3 and σ_4 represent the between-study heterogeneity for single-arm studies on each arm.

Table A1: Model parameter values specified to simulate datasets under 15 scenarios.

Scenario	n	m	l	ξ	η	σ_1	σ_2	σ_3	σ_4
S1	15	10	5	0.2	0.4	0.6	0.7	0.8	1.0
S2	10	10	10	0.2	0.4	0.6	0.7	0.8	1.0
S3	5	10	15	0.2	0.4	0.6	0.7	0.8	1.0
S4	3	12	15	0.2	0.4	0.6	0.7	0.8	1.0
S5	1	15	14	0.2	0.4	0.6	0.7	0.8	1.0
S6	15	10	5	0	0	0.6	0.7	0.8	1.0
S7	15	10	5	0.4	0.6	0.6	0.7	0.8	1.0
S8	15	10	5	0.6	0.8	0.6	0.7	0.8	1.0
S9	15	10	5	0.8	1.0	0.6	0.7	0.8	1.0
S10	15	10	5	0	0	0.6	0.7	0.1	0.3
S11	15	10	5	0	0	0.6	0.7	0.3	0.5
S12	15	10	5	0	0	0.6	0.7	0.5	0.8
S13	15	10	5	0.2	0.4	0.6	0.7	0.1	0.3
S14	15	10	5	0.2	0.4	0.6	0.7	0.3	0.5
S15	15	10	5	0.2	0.4	0.6	0.7	0.5	0.8

Appendix B - Additional results plots

We perform a sensitivity analysis by simulating data using the BPrandom method described in Section 2.1.3. Figure B.1 shows the results from the sensitivity analysis with scenarios labelled S1*-5*.

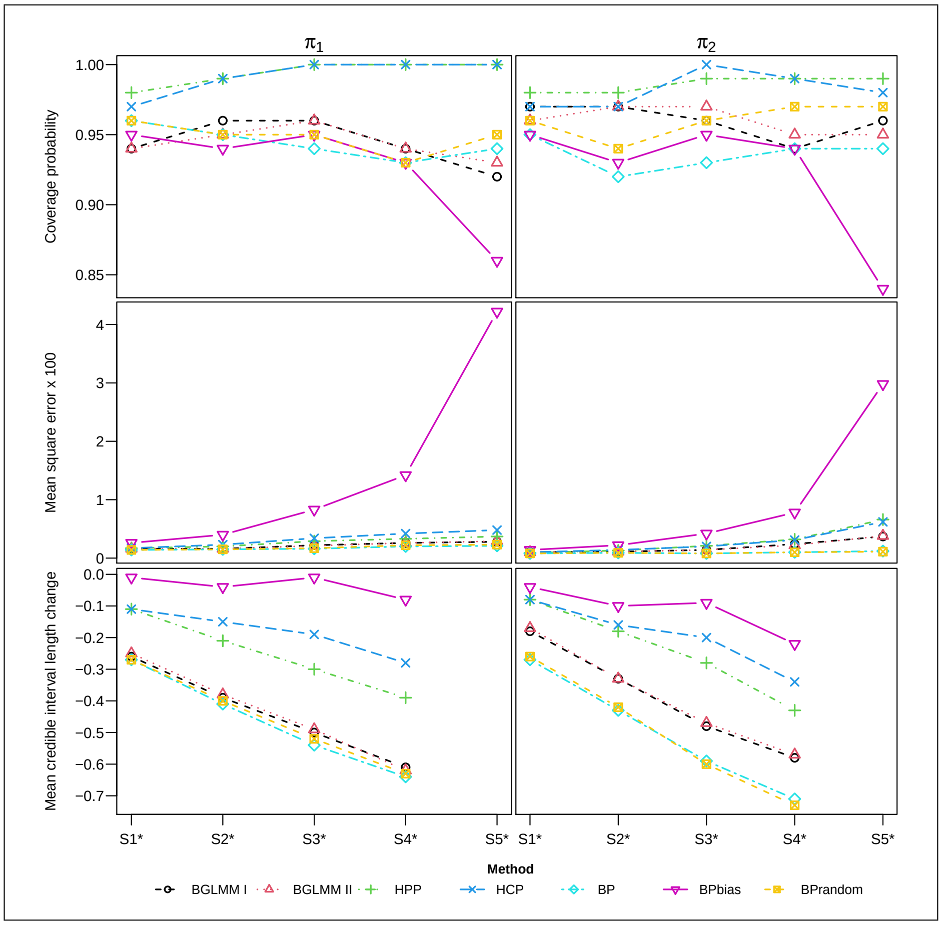

Figure B1: Coverage, MSE, and mean CrIL change for each method across scenarios S1*-5*, where data were simulated using the BPrandom method, and the number of RCTs were gradually decreased.

Appendix C - Simulation results tables

The following tables list the results for coverage, MSE, mean CrIL, and mean CrIL length change, for each of the 15 scenarios.

Table C1: Coverage, MSE, and mean CrIL change for estimands π_1 and π_2, for each method across scenarios S1-5.

Scenario	Measure	Estimand	BGLMM	BGLMM2	HPP	HCP	BP	BPbias	BPrandom
S1	Coverage	π_1	0.92	0.93	0.97	0.96	0.89	0.97	0.91
		π_2	0.95	0.94	0.96	0.95	0.90	0.93	0.97
	MSEx100	π_1	0.21	0.21	0.18	0.22	0.24	0.27	0.22
		π_2	0.15	0.14	0.14	0.17	0.14	0.23	0.12
	Length	π_1	0.17	0.17	0.19	0.19	0.16	0.22	0.16
		π_2	0.15	0.15	0.16	0.17	0.12	0.17	0.16
	Length change	π_1	-0.17	-0.17	-0.10	-0.08	-0.17	0.16	-0.19
		π_2	-0.14	-0.14	-0.11	-0.06	-0.16	0.19	-0.20
S2	Coverage	π_1	0.92	0.94	0.98	0.98	0.84	0.98	0.90
		π_2	0.95	0.95	0.97	0.96	0.89	0.96	0.97
	MSEx100	π_1	0.29	0.27	0.22	0.28	0.37	0.37	0.30
		π_2	0.18	0.18	0.17	0.21	0.16	0.35	0.14
	Length	π_1	0.19	0.20	0.22	0.24	0.17	0.29	0.17
		π_2	0.17	0.17	0.18	0.20	0.13	0.23	0.16
	Length change	π_1	-0.29	-0.26	-0.20	-0.11	-0.27	0.27	-0.27
		π_2	-0.30	-0.29	-0.23	-0.16	-0.27	0.30	-0.35
S3	Coverage	π_1	0.94	0.94	0.99	0.99	0.75	0.98	0.85
		π_2	0.97	0.98	0.99	1.00	0.86	0.97	0.95
	MSEx100	π_1	0.40	0.40	0.31	0.42	0.64	0.76	0.49
		π_2	0.23	0.22	0.22	0.30	0.21	0.71	0.18
	Length	π_1	0.22	0.23	0.27	0.33	0.18	0.42	0.20
		π_2	0.19	0.20	0.23	0.28	0.13	0.34	0.16
	Length change	π_1	-0.44	-0.41	-0.32	-0.18	-0.45	0.30	-0.41
		π_2	-0.45	-0.44	-0.33	-0.20	-0.48	0.32	-0.57
S4	Coverage	π_1	0.97	0.97	1.00	1.00	0.76	0.97	0.86
		π_2	1.00	1.00	0.99	1.00	0.84	0.94	0.93
	MSEx100	π_1	0.37	0.37	0.29	0.47	0.66	1.32	0.50
		π_2	0.24	0.24	0.27	0.37	0.25	1.33	0.21
	Length	π_1	0.25	0.24	0.31	0.39	0.18	0.49	0.20
		π_2	0.24	0.24	0.27	0.34	0.14	0.41	0.17
	Length change	π_1	-0.52	-0.55	-0.41	-0.24	-0.58	0.12	-0.55
		π_2	-0.53	-0.53	-0.45	-0.32	-0.64	0.05	-0.69
S5	Coverage	π_1	0.99	0.99	1.00	1.00	0.75	0.89	0.86
		π_2	1.00	1.00	1.00	0.96	0.77	0.85	0.92
	MSEx100	π_1	0.29	0.29	0.24	0.43	0.71	4.86	0.52
		π_2	0.26	0.26	0.45	0.61	0.39	4.30	0.28
	Length	π_1	0.26	0.26	0.35	0.58	0.20	0.59	0.21
		π_2	0.34	0.34	0.35	0.50	0.15	0.49	0.19

Table C2: Coverage, MSE, and mean CrIL change for estimands π_1 and π_2, for each method across scenarios S1*-5*.

Scenario	Measure	Estimand	BGLMM	BGLMM2	HPP	HCP	BP	BPbias	BPrandom
S1*	Coverage	π_1	0.94	0.94	0.98	0.97	0.96	0.95	0.96
		π_2	0.97	0.96	0.98	0.97	0.95	0.95	0.96
	MSEx100	π_1	0.16	0.16	0.17	0.17	0.14	0.26	0.14
		π_2	0.10	0.10	0.09	0.10	0.08	0.14	0.08
	Length	π_1	0.15	0.16	0.19	0.19	0.15	0.20	0.15
		π_2	0.13	0.14	0.15	0.15	0.11	0.15	0.11
	Length change	π_1	-0.26	-0.25	-0.11	-0.11	-0.27	-0.01	-0.27
		π_2	-0.18	-0.17	-0.08	-0.08	-0.27	-0.04	-0.26
S2*	Coverage	π_1	0.96	0.95	0.99	0.99	0.95	0.94	0.95
		π_2	0.97	0.97	0.98	0.97	0.92	0.93	0.94
	MSEx100	π_1	0.16	0.16	0.20	0.23	0.15	0.40	0.15
		π_2	0.11	0.11	0.13	0.14	0.09	0.22	0.09
	Length	π_1	0.17	0.17	0.22	0.24	0.15	0.24	0.15
		π_2	0.15	0.15	0.18	0.19	0.11	0.18	0.12
	Length change	π_1	-0.39	-0.38	-0.21	-0.15	-0.41	-0.04	-0.40
		π_2	-0.33	-0.33	-0.18	-0.16	-0.43	-0.10	-0.42
S3*	Coverage	π_1	0.96	0.96	1.00	1.00	0.94	0.95	0.95
		π_2	0.96	0.97	0.99	1.00	0.93	0.95	0.96
	MSEx100	π_1	0.22	0.21	0.29	0.34	0.16	0.83	0.17
		π_2	0.14	0.14	0.21	0.20	0.08	0.42	0.08
	Length	π_1	0.19	0.20	0.28	0.32	0.16	0.34	0.16
		π_2	0.17	0.18	0.24	0.27	0.11	0.26	0.12
	Length change	π_1	-0.50	-0.49	-0.30	-0.19	-0.54	-0.01	-0.52
		π_2	-0.48	-0.47	-0.28	-0.20	-0.59	-0.09	-0.60
S4*	Coverage	π_1	0.94	0.94	1.00	1.00	0.93	0.93	0.93
		π_2	0.94	0.95	0.99	0.99	0.94	0.94	0.97
	MSEx100	π_1	0.26	0.26	0.33	0.42	0.20	1.42	0.22
		π_2	0.24	0.22	0.32	0.31	0.10	0.78	0.10
	Length	π_1	0.20	0.20	0.31	0.37	0.16	0.42	0.17
		π_2	0.21	0.21	0.28	0.33	0.12	0.32	0.13
	Length change	π_1	-0.61	-0.62	-0.39	-0.28	-0.64	-0.08	-0.63
		π_2	-0.58	-0.57	-0.43	-0.34	-0.71	-0.22	-0.73
S5*	Coverage	π_1	0.92	0.93	1.00	1.00	0.94	0.86	0.95
		π_2	0.96	0.95	0.99	0.98	0.94	0.84	0.97
	MSEx100	π_1	0.28	0.29	0.37	0.48	0.21	4.22	0.23
		π_2	0.37	0.38	0.66	0.62	0.12	2.98	0.11
	Length	π_1	0.20	0.20	0.36	0.57	0.18	0.53	0.18
		π_2	0.30	0.30	0.35	0.50	0.13	0.42	0.15

Table C3: Coverage, MSE, and mean CrIL change for estimands π_1 and π_2, for each method across scenarios [S6, S1, S7-9].

Scenario	Measure	Estimand	BGLMM	BGLMM2	HPP	HCP	BP	BPbias	BPrandom
S6	Coverage	π_1	0.94	0.94	0.98	0.97	0.93	0.97	0.94
		π_2	0.95	0.94	0.94	0.96	0.85	0.94	0.91
	MSEx100	π_1	0.20	0.20	0.20	0.21	0.21	0.24	0.20
		π_2	0.18	0.17	0.19	0.17	0.20	0.22	0.23
	Length	π_1	0.17	0.18	0.19	0.19	0.16	0.22	0.17
		π_2	0.16	0.16	0.17	0.17	0.13	0.17	0.17
	Length change	π_1	-0.16	-0.16	-0.09	-0.08	-0.15	0.16	-0.17
		π_2	-0.08	-0.09	-0.04	-0.04	-0.11	0.19	-0.15
S1	Coverage	π_1	0.92	0.93	0.97	0.96	0.89	0.97	0.91
		π_2	0.95	0.94	0.96	0.95	0.90	0.93	0.97
	MSEx100	π_1	0.21	0.21	0.18	0.22	0.24	0.27	0.22
		π_2	0.15	0.14	0.14	0.17	0.14	0.23	0.12
	Length	π_1	0.17	0.17	0.19	0.19	0.16	0.22	0.16
		π_2	0.15	0.15	0.16	0.17	0.12	0.17	0.16
	Length change	π_1	-0.17	-0.17	-0.10	-0.08	-0.17	0.16	-0.19
		π_2	-0.14	-0.14	-0.11	-0.06	-0.16	0.19	-0.20
S7	Coverage	π_1	0.88	0.88	0.96	0.96	0.75	0.97	0.80
		π_2	0.95	0.95	0.96	0.97	0.85	0.95	0.96
	MSEx100	π_1	0.33	0.29	0.21	0.24	0.46	0.24	0.41
		π_2	0.16	0.15	0.13	0.15	0.18	0.20	0.13
	Length	π_1	0.17	0.17	0.18	0.19	0.16	0.22	0.16
		π_2	0.15	0.15	0.15	0.16	0.12	0.17	0.15
	Length change	π_1	-0.15	-0.18	-0.12	-0.07	-0.17	0.17	-0.19
		π_2	-0.15	-0.16	-0.14	-0.06	-0.18	0.20	-0.23
S8	Coverage	π_1	0.82	0.77	0.92	0.95	0.56	0.97	0.64
		π_2	0.90	0.90	0.94	0.95	0.75	0.91	0.91
	MSEx100	π_1	0.48	0.44	0.26	0.26	0.76	0.26	0.66
		π_2	0.21	0.19	0.17	0.18	0.27	0.24	0.19
	Length	π_1	0.18	0.17	0.18	0.20	0.16	0.22	0.16
		π_2	0.15	0.15	0.15	0.17	0.12	0.17	0.15
	Length change	π_1	-0.11	-0.19	-0.12	-0.05	-0.17	0.16	-0.19
		π_2	-0.16	-0.18	-0.16	-0.06	-0.20	0.19	-0.26
S9	Coverage	π_1	0.77	0.65	0.87	0.94	0.36	0.97	0.44
		π_2	0.90	0.89	0.92	0.95	0.68	0.93	0.88
	MSEx100	π_1	0.66	0.64	0.36	0.29	1.14	0.25	1.02
		π_2	0.23	0.21	0.19	0.17	0.36	0.21	0.27
	Length	π_1	0.19	0.17	0.18	0.20	0.16	0.22	0.16
		π_2	0.15	0.14	0.14	0.17	0.12	0.17	0.14
	Length change	π_1	-0.08	-0.20	-0.12	-0.04	-0.16	0.16	-0.18
		π_2	-0.15	-0.18	-0.18	-0.06	-0.20	0.19	-0.27

Table C4: Coverage, MSE, and mean CrIL change for estimands π_1 and π_2, for each method across scenarios [S10-12, S6].

Scenario	Measure	Estimand	BGLMM I	BGLMM II	HPP	HCP	BP	BPbias	BPrandom
S10	Coverage	π_1	0.98	0.98	0.98	0.98	0.88	0.82	0.92
		π_2	0.95	0.96	0.97	0.97	0.77	0.79	0.97
	MSEx100	π_1	0.03	0.03	0.18	0.15	0.13	0.32	0.12
		π_2	0.09	0.09	0.12	0.12	0.12	0.19	0.09
	Length	π_1	0.08	0.08	0.19	0.18	0.11	0.15	0.12
		π_2	0.12	0.13	0.16	0.16	0.08	0.11	0.14
	Length change	π_1	-0.62	-0.62	-0.11	-0.15	-0.42	-0.20	-0.40
		π_2	-0.29	-0.27	-0.10	-0.10	-0.44	-0.25	-0.31
S11	Coverage	π_1	0.94	0.94	0.98	0.97	0.92	0.89	0.94
		π_2	0.95	0.96	0.96	0.96	0.82	0.84	0.97
	MSEx100	π_1	0.08	0.08	0.17	0.15	0.12	0.28	0.12
		π_2	0.12	0.12	0.14	0.15	0.12	0.19	0.10
	Length	π_1	0.11	0.12	0.19	0.18	0.12	0.17	0.13
		π_2	0.14	0.14	0.16	0.16	0.09	0.12	0.14
	Length change	π_1	-0.44	-0.44	-0.10	-0.13	-0.36	-0.12	-0.35
		π_2	-0.21	-0.19	-0.08	-0.07	-0.37	-0.16	-0.28
S12	Coverage	π_1	0.94	0.94	0.97	0.96	0.94	0.94	0.94
		π_2	0.96	0.96	0.96	0.96	0.89	0.92	0.96
	MSEx100	π_1	0.14	0.14	0.18	0.17	0.15	0.25	0.14
		π_2	0.14	0.14	0.15	0.15	0.12	0.17	0.14
	Length	π_1	0.15	0.15	0.19	0.19	0.14	0.19	0.14
		π_2	0.15	0.15	0.17	0.17	0.11	0.15	0.16
	Length change	π_1	-0.29	-0.28	-0.09	-0.10	-0.26	0.01	-0.28
		π_2	-0.13	-0.13	-0.06	-0.05	-0.25	-0.01	-0.21
S6	Coverage	π_1	0.94	0.94	0.98	0.97	0.93	0.97	0.94
		π_2	0.95	0.94	0.94	0.96	0.85	0.94	0.91
	MSEx100	π_1	0.20	0.20	0.20	0.21	0.21	0.24	0.20
		π_2	0.18	0.17	0.19	0.17	0.20	0.22	0.23
	Length	π_1	0.17	0.18	0.19	0.19	0.16	0.22	0.17
		π_2	0.16	0.16	0.17	0.17	0.13	0.17	0.17
	Length change	π_1	-0.16	-0.16	-0.09	-0.08	-0.15	0.16	-0.17
		π_2	-0.08	-0.09	-0.04	-0.04	-0.11	0.19	-0.15

Table C5: Coverage, MSE, and mean CrIL change for estimands π_1 and π_2, for each method across scenarios [S13-15, S1].

Scenario	Measure	Estimand	BGLMM I	BGLMM II	HPP	HCP	BP	BPbias	BPrandom
S13	Coverage	π_1	0.27	0.27	0.98	0.97	0.61	0.87	0.73
		π_2	0.83	0.84	0.96	0.96	0.52	0.80	0.89
	MSEx100	π_1	0.28	0.27	0.17	0.19	0.35	0.28	0.26
		π_2	0.19	0.18	0.12	0.14	0.26	0.19	0.17
	Length	π_1	0.08	0.08	0.18	0.18	0.12	0.16	0.12
		π_2	0.11	0.11	0.14	0.16	0.08	0.11	0.13
	Length change	π_1	-0.62	-0.63	-0.13	-0.13	-0.41	-0.20	-0.40
		π_2	-0.36	-0.37	-0.20	-0.12	-0.46	-0.25	-0.36
S14	Coverage	π_1	0.72	0.71	0.97	0.96	0.70	0.89	0.79
		π_2	0.89	0.90	0.96	0.94	0.66	0.83	0.92
	MSEx100	π_1	0.24	0.23	0.17	0.19	0.33	0.28	0.25
		π_2	0.16	0.15	0.13	0.16	0.22	0.20	0.15
	Length	π_1	0.11	0.11	0.18	0.18	0.12	0.17	0.13
		π_2	0.13	0.13	0.15	0.16	0.09	0.13	0.14
	Length change	π_1	-0.46	-0.47	-0.13	-0.12	-0.36	-0.12	-0.36
		π_2	-0.29	-0.28	-0.18	-0.10	-0.40	-0.16	-0.32
S15	Coverage	π_1	0.88	0.88	0.97	0.96	0.81	0.94	0.86
		π_2	0.96	0.96	0.97	0.97	0.81	0.90	0.96
	MSEx100	π_1	0.22	0.20	0.18	0.21	0.29	0.26	0.24
		π_2	0.13	0.12	0.12	0.14	0.16	0.18	0.12
	Length	π_1	0.14	0.14	0.18	0.19	0.14	0.19	0.14
		π_2	0.14	0.14	0.15	0.17	0.10	0.15	0.15
	Length change	π_1	-0.30	-0.30	-0.12	-0.10	-0.27	0.01	-0.29
		π_2	-0.19	-0.19	-0.14	-0.07	-0.30	-0.01	-0.27
S1	Coverage	π_1	0.92	0.93	0.97	0.96	0.89	0.97	0.91
		π_2	0.95	0.94	0.96	0.95	0.90	0.93	0.97
	MSEx100	π_1	0.21	0.21	0.18	0.22	0.24	0.27	0.22
		π_2	0.15	0.14	0.14	0.17	0.14	0.23	0.12
	Length	π_1	0.17	0.17	0.19	0.19	0.16	0.22	0.16
		π_2	0.15	0.15	0.16	0.17	0.12	0.17	0.16
	Length change	π_1	-0.17	-0.17	-0.10	-0.08	-0.17	0.16	-0.19
		π_2	-0.14	-0.14	-0.11	-0.06	-0.16	0.19	-0.20

Appendix D - WinBUGS code

Begg & Pilote (BP) original method

```
model{
  # Model for RCTs.
  for(i in 1:N1){
    r1[i] ~ dbin(p1[i], totaln1[i])
    r2[i] ~ dbin(p2[i], totaln2[i])
    p1[i] <- phi(theta[i])
    p2[i] <- phi(theta[i] + delta)
  }

  # Model for single-arm studies on treatment 1.
  for(j in 1:N2){
    r3[j] ~ dbin(p3[j], totaln3[j])
    p3[j] <- phi(theta[j + N1])
  }

  # Model for single-arm studies on treatment 2.
  for(j in 1:N3){
    r4[j] ~ dbin(p4[j], totaln4[j])
    p4[j] <- phi(theta[j + N1 + N2] + delta)
  }

  # Model to combine effects.
  for (j in 1:(N1 + N2 + N3)){
    theta[j] ~ dnorm(mu.theta, tau.theta)
  }

  # Priors.
  mu.theta ~ dnorm(0, 0.001)
  delta ~ dnorm(0, 0.001)
  tau.theta ~ dgamma(0.001, 0.001)
  var.theta <- 1 / tau.theta

  # Estimands.
  pi1 <- phi(mu.theta / sqrt(1 + var.theta))
  pi2 <- phi((mu.theta + delta) / sqrt(1 + var.theta))
  OR <- (pi2 / (1 - pi2)) / (pi1 / (1 - pi1))
}
```

Begg & Pilote method with bias-adjustment (BPbias)

```
model{
  # Model for RCTs.
  for(i in 1:N1){
    r1[i] ~ dbin(p1[i], totaln1[i])
    r2[i] ~ dbin(p2[i], totaln2[i])
    p1[i] <- phi(theta[i])
    p2[i] <- phi(theta[i] + delta)
  }

  # Model for single-arm studies on treatment 1.
  for(j in 1:N2){
    r3[j] ~ dbin(p3[j], totaln3[j])
    p3[j] <- phi(theta[j + N1] + xi)
  }

  # Model for single-arm studies on treatment 2.
  for(j in 1:N3){
    r4[j] ~ dbin(p4[j], totaln4[j])
    p4[j] <- phi(theta[j + N1 + N2] + delta + eta)
  }

  # Model to combine effects.
  for (j in 1:(N1 + N1 + N3)){
    theta[j] ~ dnorm(mu.theta, tau.theta)
  }

  # Priors.
  mu.theta ~ dnorm(0, 0.001)
  delta ~ dnorm(0, 0.001)
  tau.theta ~ dgamma(0.001, 0.001)
  var.theta <- 1 / tau.theta
  xi ~ dnorm(0, 0.001)
  eta ~ dnorm(0, 0.001)

  # Estimands.
  pi1 <- phi(mu.theta / sqrt(1 + var.theta))
  pi2 <- phi((mu.theta + delta) / sqrt(1 + var.theta))
  OR <- (pi2 / (1 - pi2)) / (pi1 / (1 - pi1))
}
```

Begg & Pilote method with random effects (BPrandom)

```
model{
  # Model for RCTs.
  for(i in 1:N1){
    r1[i] ~ dbin(p1[i], totaln1[i])
    r2[i] ~ dbin(p2[i], totaln2[i])
    p1[i] <- phi(theta[i])
    p2[i] <- phi(theta[i] + delta[i])
  }

  # Model for single-arm studies on treatment 1.
  for(j in 1:N2){
    r3[j] ~ dbin(p3[j], totaln3[j])
    p3[j] <- phi(theta[j + N1])
  }

  # Model for single-arm studies on treatment 2.
  for(j in 1:N3){
    r4[j] ~ dbin(p4[j], totaln4[j])
    p4[j] <- phi(theta[j + N1 + N2] + delta[j + N1])
  }

  # Model to combine effects.
  for (j in 1:(N1 + N2 + N3)){
    theta[j] ~ dnorm(mu.theta, tau.theta)
  }

  for (j in 1:(N1 + N3)){
    delta[j] ~ dnorm(mu.delta, tau.delta)
  }

  # Priors.
  mu.theta ~ dnorm(0, 0.001)
  mu.delta ~ dnorm(0, 0.001)
  tau.theta ~ dgamma(0.001, 0.001)
  tau.delta ~ dgamma(0.001, 0.001)
  var.theta <- 1 / tau.theta
  var.delta <- 1 / tau.delta

  # Estimands.
  pi1 <- phi(mu.theta / sqrt(1 + var.theta))
  pi2 <- phi((mu.theta + mu.delta) / sqrt(1 + var.theta + var.delta))
  OR <- (pi2 / (1 - pi2)) / (pi1 / (1 - pi1))
}
```

Bivariate generalised mixed-effects model (BGLMM) I

```
model{
  # Model for RCTs.
  for(i in 1:N1){
    r1[i] ~ dbin(p1[i], totaln1[i])
    r2[i] ~ dbin(p2[i], totaln2[i])
    p1[i] <- phi(mu1 + vi[i, 1])
    p2[i] <- phi(mu2 + vi[i, 2])
    vi[i, 1:2] ~ dmnorm(mn[1:2], T[1:2, 1:2])
  }

  # Model for single-arm studies on treatment 1.
  for(j in 1:N2){
    r3[j] ~ dbin(p3[j], totaln3[j])
    p3[j] <- phi(mu1 + s[j])
    s[j] ~ dnorm(0, tau1)
  }

  # Model for single-arm studies on treatment 2.
  for(j in 1:N3){
    r4[j] ~ dbin(p4[j], totaln4[j])
    p4[j] <- phi(mu2 + t[j])
    t[j] ~ dnorm(0, tau2)
  }

  # Priors.
  mu1 ~ dnorm(0, 0.001)
  mu2 ~ dnorm(0, 0.001)
  T[1:2,1:2] ~ dwish(R[1:2, 1:2], 2)
  tau1 ~ dgamma(0.001, 0.001)
  tau2 ~ dgamma(0.001, 0.001)

  # Estimands.
  invT[1:2, 1:2] <- inverse(T[, ])
  var1 <- invT[1, 1]
  var2 <- invT[2, 2]
  pi1 <- phi(mu1 / sqrt(1 + var1))
  pi2 <- phi(mu2 / sqrt(1 + var2))
  OR <- (pi2 / (1 - pi2)) / (pi1 / (1 - pi1))
}
```

Bivariate generalised mixed-effects model (BGLMM) II

Part 1

```
model{
  # Model for single-arm studies on treatment 1.
  for(j in 1:N2){
    r3[j] ~ dbin(p3[j], totaln3[j])
    p3[j] <- phi(mu3 + s[j])
    s[j] ~ dnorm(0, tau1)
  }

  # Model for single-arm studies on treatment 2.
  for(j in 1:N3){
    r4[j] ~ dbin(p4[j], totaln4[j])
    p4[j] <- phi(mu4 + t[j])
    t[j] ~ dnorm(0, tau2)
  }

  # Priors.
  mu3 ~ dnorm(0, 0.001)
  mu4 ~ dnorm(0, 0.001)
  tau1 ~ dgamma(0.001, 0.001)
  tau2 ~ dgamma(0.001, 0.001)
}
```

Part 2

```
model{
  # Model for RCTs.
  for(i in 1:N1){
    r1[i] ~ dbin(p1[i], totaln1[i])
    r2[i] ~ dbin(p2[i], totaln2[i])
    p1[i] <- phi(mu1 + vi[i, 1])
    p2[i] <- phi(mu2 + vi[i, 2])
    vi[i, 1:2] ~ dmnorm(mn[1:2], T[1:2, 1:2])
  }

  # Priors.
  mu3prec <- 1 / (mu3sd * mu3sd)
  mu4prec <- 1 / (mu4sd * mu4sd)
  mu1 ~ dnorm(mu3med, mu3prec)
  mu2 ~ dnorm(mu4med, mu4prec)
  T[1:2,1:2] ~ dwish(R[1:2, 1:2], 2)

  # Estimands.
  invT[1:2, 1:2] <- inverse(T[, ])
  var1 <- invT[1, 1]
  var2 <- invT[2, 2]
  pi1 <- phi(mu1 / sqrt(1 + var1))
  pi2 <- phi(mu2 / sqrt(1 + var2))
  OR <- (pi2 / (1 - pi2)) / (pi1 / (1 - pi1))
}
```

Hierarchical power prior (HPP)

```
model{
  # Model for RCTs.
  for(i in 1:N1){
    r1[i] ~ dbin(p1[i], totaln1[i])
    r2[i] ~ dbin(p2[i], totaln2[i])
    p1[i] <- phi(mu1 + vi[i, 1])
    p2[i] <- phi(mu2 + vi[i, 2])
    vi[i, 1:2] ~ dmnorm(mn[1:2], T[1:2, 1:2])
  }
  # Model for single-arm studies on treatment 1.
  for(j in 1:N2){
    z1[j] ~ dpois(phi1[j])
    phi1[j] <- a1 * -(r3[j] * log(p3[j]) +
        (totaln3[j] - r3[j]) * log(1 - p3[j])) + const
    p3[j] <- phi(mu1 + s[j])
    s[j] ~ dnorm(0, tau1)
  }
  # Model for single-arm studies on treatment 2.
  for(j in 1:N3){
    z2[j] ~ dpois(phi2[j])
    phi2[j] <- a2 * -(r4[j] * log(p4[j]) +
        (totaln4[j] - r4[j]) * log(1 - p4[j])) + const
    p4[j] <- phi(mu2 + t[j])
    t[j] ~ dnorm(0, tau2)
  }

  # Priors.
  mu1 ~ dnorm(0, 0.001)
  mu2 ~ dnorm(0, 0.001)
  T[1:2,1:2] ~ dwish(R[1:2, 1:2], 2)
  tau1 ~ dgamma(0.001, 0.001)
  tau2 ~ dgamma(0.001, 0.001)
  a1 ~ dbeta(10, 1)
  a2 ~ dbeta(10, 1)

  # Estimands.
  invT[1:2, 1:2] <- inverse(T[, ])
  var1 <- invT[1, 1]
  var2 <- invT[2, 2]
  pi1 <- phi(mu1 / sqrt(1 + var1))
  pi2 <- phi(mu2 / sqrt(1 + var2))
  OR <- (pi2 / (1 - pi2)) / (pi1 / (1 - pi1))
}
```

Hierarchical commensurate prior (HCP)

```
model{
  # Model for RCTs.
  for(i in 1:N1){
    r1[i] ~ dbin(p1[i], totaln1[i])
    r2[i] ~ dbin(p2[i], totaln2[i])
    p1[i] <- phi(mu1 + vi[i, 1])
    p2[i] <- phi(mu2 + vi[i, 2])
    vi[i, 1:2] ~ dmnorm(mn[1:2], T[1:2, 1:2])
  }

  # Model for single-arm studies on treatment 1.
  for(j in 1:N2){
    r3[j] ~ dbin(p3[j], totaln3[j])
    p3[j] <- phi(mu3 + s[j])
    s[j] ~ dnorm(0, tau1)
  }

  # Model for single-arm studies on treatment 2.
  for(j in 1:N3){
    r4[j] ~ dbin(p4[j], totaln4[j])
    p4[j] <- phi(mu4 + t[j])
    t[j] ~ dnorm(0, tau2)
  }

  # Priors.
  mu1 ~ dnorm(mu3, bias1)
  mu2 ~ dnorm(mu4, bias2)
  mu3 ~ dnorm(0, 0.001)
  mu4 ~ dnorm(0, 0.001)
  T[1:2,1:2] ~ dwish(R[1:2, 1:2], 2)
  tau1 ~ dgamma(0.01, 0.01)
  tau2 ~ dgamma(0.01, 0.01)
  bias1 ~ dgamma(0.01, 0.01)
  bias2 ~ dgamma(0.01, 0.01)

  # Estimands.
  invT[1:2, 1:2] <- inverse(T[, ])
  var1 <- invT[1, 1]
  var2 <- invT[2, 2]
  pi1 <- phi(mu1 / sqrt(1 + var1))
  pi2 <- phi(mu2 / sqrt(1 + var2))
  OR <- (pi2 / (1 - pi2)) / (pi1 / (1 - pi1))
}
```
